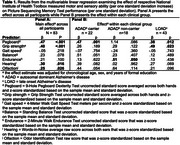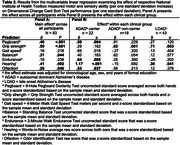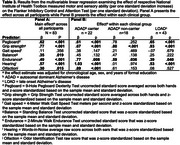# Associations between motor and sensory abilities with memory and executive function in Latinos: Comparison between autosomal dominant and late‐onset Alzheimer's disease

**DOI:** 10.1002/alz70857_103357

**Published:** 2025-12-25

**Authors:** Andrew J. Petkus, Bryan Rowe, Lucy Montoya, Abhay P Sagare, John M Ringman

**Affiliations:** ^1^ University of Southern California, Los Angeles, CA, USA; ^2^ Department of Neurology, Keck School of Medicine at USC, Los Angeles, CA, USA; ^3^ Zilkha Neurogenetic Institute, Keck School of Medicine, University of Southern California, Los Angeles, CA, USA; ^4^ Alzheimer's Disease Research Center, Keck School of Medicine, University of Southern California, Los Angeles, CA, USA

## Abstract

**Background:**

Declining motor and sensory abilities are risk factors for Alzheimer's disease (AD). However, their relationships with cognitive performance remain understudied in Latino populations and individuals with autosomal dominant AD (ADAD), who experience fewer confounding factors than those at risk for late‐onset AD (LOAD). We examined heterogeneity in the associations between motor ability, hearing, and olfaction with episodic memory and executive performance in a predominantly Latino sample at risk for either ADAD or LOAD.

**Method:**

This cross‐sectional study included 83 Latinos who completed the motor, sensation, and cognitive batteries from the National Institute of Health Toolbox. Participants were in one of three clinical groups: (1) carriers of the ADAD mutation (*n* = 22; mean age 37.6 ± 11.8), (2) were at 50% risk for inheriting the mutation but were noncarriers (*n* = 18; mean age 34.7 ± 10.4), or (3) older Latino's at risk for or having LOAD (*n* = 43; mean age 67.3 ± 8.3). Multivariable linear regressions were used to examine the associations between motor and sensory abilities with episodic memory (Picture Sequence Memory Test), cognitive flexibility (Dimensional Change Card Sort), and inhibition (Flanker Inhibitory Control and Attention) performance. We then examined the effect moderation by clinical group. All regressions were adjusted for age, sex, and education.

**Result:**

Across all participants, poorer pegboard performance, grip strength, and hearing abilities (per one standard deviation) were associated with worse performance on memory (Table 1), cognitive flexibility (Table 2), and inhibition (Table 3) tests. Balance and olfaction were also associated with flexibility and inhibition. Associations between hearing with flexibility and inhibition were of significantly larger magnitude (*p* < .05) in ADAD mutation carriers compared to the other clinical groups. Associations between other motor and sensory assessments and episodic memory, flexibility, and inhibition were not significantly different across clinical groups. Similar effects were observed after excluding individuals with a Clinical Dementia Rating (CDR) of 1.0 or higher.

**Conclusion:**

Poorer hearing, reduced fine motor skills, and diminished upper body strength are linked to worse memory and executive function in Latinos with ADAD or with or at risk for LOAD. The neuropathological processes associated with Alzheimer's Disease may adversely affect motor skills and auditory capabilities.